# Announcement: The Royal Society of Chemistry, Dalton Division, Dalton Discussion No. 2, Bioinorganic Chemistry

**DOI:** 10.1155/MBD.1997.293

**Published:** 1997


					THE ROYAL SOCIETY OF CHEMISTRY
DALTON DIVISION
DALTON DISCUSSION NO. 2
BIOINORGANIC CHEMISTRY
,-n5 Sep.tember 1997
University of East lia/Nitrogen Fixation Laboratory, UK
Sessions and Keynote Speakers
Oxomolybdenum and Oxotungsten Enzymes
Session Leader: Dave Garner
A Crystallographic View of the Molybdenum-Cofactor
Doug Rees- Caltech, USA
Molybdenum Enzymes: Learning from Chemistry
Ed Stiefel- Exxon Research, USA
Small Molecule Activation by Metalloproteins
Session Leader: Roger Thorneley
Principles of Small Molecule Activation 10y Metalloenzymes as Exemplified by the $olulole Metlane
Monooxygenases
Ann Valentine- MIT, USA
Oxygen Activation at Nonheme Diiron Active Sites in Biology: Lessons from Model Complexes
Larry Que- University of Minnesota, USA
Biomineralisation
Session Leaders: Annie Powell and Geoff Moore
Biomineralization: the Form(id)able Part of Bioinorganic Chemistry!
Steve Mann University of Bath, UK
Inorganic Life. Morphogenesis of Natural Form
Geoff Ozin University of Toronto, Canada
Mixed-valance Metal Clusters in Biology
Session Leader: Andrew Thomson
EPR Study of the S 1/2 Ground State of Radiolysis-generated Mn(lll) Mn(IV)3 form of
[Mn(IV)406(bpy)6]4+. Comparison with the Photosynthetic Oxygen Evolving Complex
Genevieve Blondin University of Paris Sud, France
Manganese Clusters: A Common Ground for Photosynthesis, Quantum Tunnelling of
Magnetizaton and Colossal Magnetoresistance
Dante Gatteschi- University of Florence, Italy
Metals in Medicine
Session Leader: Peter Sadler
Hydrogen Bonding of Nucleobases: Effects of Metal Ion Binding
Bernhardt Lippert University of Dortmund, Germany
Intramolecular Migration of Coordinated Platinum from a Sulfur to N7 in the Nucleopeptide Met-d
(TpG)
Jan Reedijk- University of Leiden, The Netherlands
Contacts:
Dr A K Powell
School of Chemical Sciences
UEA
Norwich NR4 7TJ, UK
E-mail: A.Powell@uea.ac.uk
Dr. John F Gibson
"Bioinorganic Chemistry" Discussion
The Royal Society of Chemistry
Burlington House, London W1V 0BN, UK
Tel: +44 (0) 171 437 8656
Fax: +44 (0) 171 734 1227
E-mail: conferences@rsc.org
293

				

